# Real-world evaluation of ceftriaxone-related safety events: a stewardship call to action

**DOI:** 10.1017/ash.2025.10258

**Published:** 2025-12-22

**Authors:** Kristen Paciullo, Sujit Suchindran, Leila S. Hojat, Benjamin Albrecht, Sarah B. Green, K. Ashley Jones, Daniel J. Rogers, Trinh P. Vu, Lucy S. Witt

**Affiliations:** 1 Pharmacy Department, Emory Healthcare, Atlanta, GA, USA; 2 Division of Infectious Diseases, Department of Medicine, Emory University School of Medicine, Atlanta, GA, USA

## Abstract

A ceftriaxone safety alert prompted a review of rapid response and cardiac arrest events in relation to the timing of intravenous cephalosporin administrations across a large health system. Despite high utilization, we found a low rate of significant ceftriaxone-related adverse events with a similar incidence as other intravenous cephalosporins.

## Introduction

Ceftriaxone is a third-generation cephalosporin recommended as an empiric therapy option in many clinical practice guidelines.^1,2^ Ceftriaxone is used extensively due to its broad spectrum of activity, favorable pharmacokinetics, ease of dosing, limited drug-drug interactions, and high tolerability. Adverse effects associated with ceftriaxone are generally mild to moderate, with most reported being gastrointestinal related.^2^ In January 2025, the Alabama Department of Public Health issued an alert after receiving reports of 11 cases of potential adverse effects associated with ceftriaxone.^3^ In February 2025, the Georgia Department of Public Health (GA DPH) issued a similar health alert with approximately 10 cases reported.^4^ In collaboration with health departments across the United States, the Centers for Disease Control and Prevention (CDC) started an investigation to identify and characterize serious adverse events associated with ceftriaxone exposure. Since no manufacturer or lot number has been associated with these events, and no causal link to ceftriaxone has been established, a comprehensive review of significant events occurring after intravenous (IV) cephalosporin use across the inpatient setting was performed at our multi-hospital healthcare system to (1) identify ceftriaxone-related safety events as recommended by GA DPH and the CDC and (2) to characterize the rates of ceftriaxone-related events compared to other cephalosporins.

## Methods

As part of the investigation, the CDC asked healthcare providers to report cases dating back to September 1, 2024, that met the following criteria: occurring within six hours after receipt of injectable ceftriaxone in a non-intensive care unit (ICU) setting that resulted in death or required cardiopulmonary resuscitation (CPR) and was not attributed by the provider(s) to a cause other than ceftriaxone administration.^5^ A documentation tool within the electronic health record (EHR), “Code Narrator” was utilized to retrospectively identify significant events occurring within four hours after administration of an IV cephalosporin (cefazolin, cefuroxime, ceftriaxone, ceftazidime, cefepime) from October 1, 2022, to June 30, 2025, across our healthcare system comprising seven hospitals located in urban and suburban settings in Georgia. Code Narrator identifies rapid response events; those where a dedicated team responds to an acutely deteriorating patient, in addition to cases requiring CPR. Of those identified, a manual chart review was performed. While we excluded events which occurred in an ICU to align with the CDC case criteria, those included differed from the CDC definition in terms of timing of event (4 vs 6 h) and time period evaluated (October 1, 2022 vs September 1, 2024) and was applied to both the ceftriaxone and non-ceftriaxone cephalosporin group. Patient characteristics extracted from the EHR included hospital, department in which event occurred, type of administration, event time, and time from administration of cephalosporin to event. Additional patient characteristics collected via chart review included type of event (rapid response vs cardiac arrest) and presence of documented beta-lactam allergy. Concomitant administration of a proton pump inhibitor (PPI) and length of QTc interval on date of event was collected only for the ceftriaxone group. Frequency of rapid response and cardiac arrest events per number of administrations for each cephalosporin were calculated. Details of events specifically documented as related to a cephalosporin by the treating providers in the medical record were obtained.

## Data analysis

Descriptive analysis was performed by calculating frequencies and percentages. Chi-squared or Fisher’s Exact tests were performed to compare categorical variables and evaluate associations, depending on sample size and expected frequency distributions in the contingency table.

## Results

During the period evaluated, 90 events were identified in patients who received ceftriaxone and 121 events for cefazolin, cefuroxime, ceftazidime, and cefepime combined (non-ceftriaxone group). A description of events including location, type of administration, and patient characteristics is included in Table 1. The location of the event was the only statistically significant difference noted between the two groups. While most patients included did not have a documented beta-lactam allergy, no differences were noted between those with beta-lactam allergies and those without (*p* = .08).

**Table 1.** Description of significant events occurring within four hours of receipt of an IV cephalosporin in a non-ICU setting

	Ceftriaxone group (*n* = 90)		Non-ceftriaxone group (*n* = 121)		*P*-value
**Location of event, *n* (%)**					
**Emergency department**	38 (42.2)		16 (13.2)		<.01
**Imaging**	11 (12.2)		8 (6.6)		.22
**Wards**	39 (43.3)		60 (49.6)		.01
**OR/Procedural**	2 (2.2)		37 (30.6)		<.01
**Administration, *n* (%)**					
**IV Push**	89 (98.9)		116 (95.9)		.24
**Intermittent IV Infusion**	1 (1.1)		5 (4.1)		
**Patient characteristics**					
Beta lactam allergy, yes, *n* (%)^*^	12 (13.3)		24 (19.8)		.21
Penicillin, *n* (%)	11 (91.7)		22 (91.7)		.81
Cephalosporin, *n* (%)	1 (8.3)		3 (12.5)		
Concomitant PPI^**^ use, yes, *n* (%)	20 (22.2)		--		--
**QTc Interval, mean (SD)^**			--		--
Without concomitant PPI	460 (56.5)		--		--
With concomitant PPI	473 (45.5)		--		--
**Patient characteristics based on type of event**					
**Cardiac arrest, *n* (%)**	**Ceftriaxone group (*n* = 59)**		**Non-ceftriaxone group (*n* = 70)**		
	Yes	No	Yes	No	
Beta lactam allergy	5 (8.5)	54 (91.5)	13 (18.6)	57 (81.4)	.08
Concomitant PPI use	12 (20.3)	47 (79.7)	--	--	--
**Rapid response, *n* (%)**	**Ceftriaxone group (*n* = 31)**		**Non-ceftriaxone group (*n* = 51)**		
	Yes	No	Yes	No	
Beta lactam allergy	7 (22.6)	24 (77.4)	11 (21.6)	40 (78.4)	.91
Concomitant PPI use	8 (25.8)	23 (74.2)	--	--	--

* One patient had both penicillin and cephalosporin allergies documented.

** PPI was pantoprazole for all patients evaluated. ^QTc was not available on the date of event for 7 patients receiving PPI and 19 patients not receiving PPI.

A higher percentage of events in the ceftriaxone group were cardiac arrests, while events were more frequently classified as rapid responses in the non-ceftriaxone group, though these differences were not significant (Table 2). Frequency of events between the ceftriaxone and non-ceftriaxone groups were similar with 0.047% of ceftriaxone administrations being associated with an event compared to 0.041% for non-ceftriaxone cephalosporins (*p* = 0.33).

**Table 2.** Type and frequency of events relative to cephalosporin utilization in non-ICU settings

	Ceftriaxone group	Non-ceftriaxone group	*P*-value
**Number of Events**	**N = 90**	**N = 121**	
**Cardiac arrest, *n* (%)**	59 (65.6)	70 (57.9)	.26
**Rapid response, *n* (%)**	31 (34.4)	51 (42.1)	
Altered mental status	1 (3.2)	9 (17.6)	.03
Arrhythmia	8 (25.8)	7 (13.7)	.39
Chest pain	0	2 (3.9)	.33
Hyperglycemia	1 (3.2)	0	1
Hypotension	4 (12.9)	12 (23.5)	.11
Hypoxia	11 (35.5)	18 (35.3)	.58
Seizure	4 (12.9)	1 (2.0)	1
Stroke	2 (6.5)	2 (3.9)	1
**Number of non-ICU inpatient IV cephalosporin administrations**			
**Ceftriaxone**	189,777	--	
**Non-ceftriaxone (total)**		292,071	
Cefazolin	--	126,629	
Cefuroxime	--	2,682	
Ceftazidime	--	40,597	
Cefepime	--	122,163	
**Frequency of events** ^*^			
**All events**	.047%	.041%	.33
Cardiac arrest	.031%	.024%	.14
Rapid response	.016%	.017%	.77

* Frequency of events = (# of events/# of administrations) x 100.

Of the cardiac arrests which occurred following the administration of an IV cephalosporin, three events were attributed to ceftriaxone by the clinical team in the clinical record, and 0 events were attributed to a cephalosporin in the non-ceftriaxone group.

## Discussion

This study compared rapid response and cardiac arrest events following the administration of ceftriaxone versus other IV cephalosporins in a large healthcare system. High ceftriaxone utilization was noted, and significant events were low and similar to event rates for non-ceftriaxone cephalosporins. The three cardiac events attributed to ceftriaxone occurred in patients with multiple co-morbidities, within 15 mins of administration. While unable to determine causality, given the presentations, “Kounis syndrome,” should be considered. Described as a complex, allergy – induced cause of acute coronary syndromes, this syndrome has been reported following administration of beta lactams, with the majority of cases occurring within 30 mins of administration.^6^ While most commonly described with penicillins, cases involving ceftriaxone have been reported.^7^ Based on previous literature identifying an increased risk of cardiovascular events related to QTc prolongation with concomitant lansoprazole and ceftriaxone use,^8^ PPI use in the ceftriaxone group was also evaluated. Patients who were on a PPI received pantoprazole, as opposed to lansoprazole, and no significant differences in QTc interval were noted. The method of administration of ceftriaxone (IV push vs intermittent infusion) and impact on risk of adverse events has also been questioned, however not robustly evaluated in literature. When administered over 3—5 mins, ceftriaxone administered via IV push has been found to be safe and has advantages related to faster time to medication administration, decreased length of stay, and less waste associated with mini bags and tubing.^9,10^ We were unable to comment on the risk of IV push versus infusion given the limited number of infusions employed in our healthcare system.

## Limitations

Our study is a retrospective evaluation of narrowly defined adverse events. Given the retrospective nature, we could not thoroughly investigate alternative causes of reported events. Our hospital system serves almost exclusively adult patients, making our data potentially not generalizable to children. Additionally, we limited our analysis to events occurring within four hours of cephalosporin administration, rather than the 6-hour window defined by the CDC case criteria. We believe this is more likely to capture adverse events due to drug administration and reduce inclusion of unrelated events.

## Conclusion

Due to the low frequency of events and comparable rates to other cephalosporins, avoidance of ceftriaxone was not recommended by our antimicrobial stewardship team. Given the broad use of ceftriaxone and a small number of cases related to significant adverse effects identified, the public health alert issued by the CDC and state departments can be challenging for healthcare systems to address. As there are no recommendations from the CDC to withhold or recommend alternatives to ceftriaxone while the investigation is ongoing, stewardship teams must employ their own strategies to help guide optimal antibiotic use while trying to minimize unintended consequences.
